# A Rare Case of Stroke Secondary to Iron Deficiency Anemia in a Young Female Patient

**DOI:** 10.1155/2017/1684631

**Published:** 2017-02-28

**Authors:** Kavitha Gopalratnam, Kevin Andrew Woodson, Jigarkumar Rangunwala, Kanaga Sena, Manisha Gupta

**Affiliations:** Bridgeport Hospital-Yale New Haven Health, 267 Grant St, Bridgeport, CT 06610, USA

## Abstract

Ischemic strokes occur when there is a sudden obstruction of an artery supplying blood flow to an area of the brain, leading to a focal neurological deficit. Strokes can be thrombotic or embolic in etiology and are associated with underlying conditions such as hypertension and atherosclerosis. Possible etiologies of strokes include cardioembolic disease, hematologic disorders, connective tissue disorders, and substance abuse or can be cryptogenic. Most stroke cases are seen in patients over 65 years of age. However, about one-fourth of strokes occur in young adults. Iron deficiency anemia (IDA) has been described as a known cause for strokes in children, but very few case reports describe this association in adults. We describe a 20-year-old female who presented with sudden onset left side weakness. Magnetic Resonance Imaging (MRI) of the brain demonstrated ischemic infarctions. Patient was also found to be severely anemic. Patient had a thorough work-up including Magnetic Resonance Angiography (MRA) of the brain, echocardiogram, and an extensive screen for thrombophilia disorders. This, however, did not demonstrate a clear etiology. As it has been suggested that IDA is a potential cause for stroke, it is possible the stroke in this young patient was attributable to severe IDA.

## 1. Introduction

Ischemic strokes are a common problem primarily in patients above 65 years of age [1]. In this case, we describe a young patient who suffered an ischemic stroke at 20 years of age. The patient's only comorbidity was severe IDA. This case is interesting as IDA is an etiology for stroke which is typically only seen in pediatric patients [2]. The case is significant as it suggests IDA as a possible etiology for stroke in young adults. IDA is not commonly associated with stroke in the minds of most clinicians. As IDA is a common condition in young adults, we suggest that prompt diagnosis and treatment of IDA should be a priority for young patients who present with stroke when all other more common etiologies are ruled out.

## 2. Case Presentation

A 20-year-old female presented to the hospital after being involved in a motor vehicle accident. The accident was preceded by a sensation of dizziness with blurring of vision and brief loss of consciousness. Her co-passengers, who witnessed the event, denied noticing any seizure-like activity. She regained consciousness within a few minutes, at which time she was alert and responding appropriately. Upon arrival to the hospital, she was noted to have some chest pain where the air bag had deployed but did not have any signs of significant trauma. She endorsed continued dizziness, blurring of vision, and photophobia. Also, she attested to some weakness in her left side involving her arms and legs. On physical exam, she demonstrated decreased motor strength in her left upper extremity but otherwise intact neurological exam. An initial head CT was negative for any ischemic changes. Laboratory tests revealed severe anemia with hemoglobin of 5.8 mg/dL and platelet count of 564, suggestive of reactive thrombocytosis. Patient also was found to have serum iron 20 *μ*g/dL, TIBC 481 *μ*g/dL, iron saturation 6%, and serum ferritin 39 ng/mL. MCV was 64 fL/cell and RDW was 18.2%. Other laboratory tests including white blood count, chemistry panel, lipid panel, urea, and creatinine were within normal limits. History revealed menorrhagia with multiple evaluations for near syncope and dizziness. However, she did not have active bleeding at the time of presentation. Her clinical course was further complicated by low grade fevers, confusion, lethargy, and changes in her mental status. She demonstrated significant neurological findings on physical exam with worsening left upper extremity strength, bilateral dysmetria, and a positive Romberg sign. A lumbar puncture did not reveal any infectious causes of her symptoms. MRI at the time revealed bilateral cerebellar and left occipital lobe nonhemorrhagic infarcts. An MRA of the brain ruled out arterial dissection or thrombus formation. She also had an echocardiogram, which did not show any signs of PFO or interatrial shunt with agitated saline study. A thorough work-up for underlying thrombophilia was unrevealing including protein S and C deficiency, lupus anticoagulant, anti-cardiolipin antibody, Factor V Leiden mutation, ANA positivity, antithrombin III deficiency, Vitamin B12 level, homocysteine level, hemoglobin electrophoresis, and rapid plasma reagin. The patient received multiple blood transfusions and showed good response with improvement in her hemoglobin. Additionally, patient received intravenous iron infusion and ferrous sulfate 325 mg TID was initiated to help correct the IDA. She showed improvement in neurological function over a period of two weeks with concurrent inpatient physical therapy. Patient was seen in the resident's continuity clinic in the following months with resultant improvement in iron saturation and serum ferritin. No additional neuroimaging was performed.

## 3. Discussion

IDA accounts for about 50% of all anemia cases worldwide. It has a prevalence of about 2–4% in nonpregnant women 12–49 years of age and about 2–5% prevalence in men and postmenopausal women [3, 4]. IDA can be caused by poor dietary intake, poor absorption of iron, or bleeding. Possible sources of blood loss include the gastrointestinal and genitourinary systems.

Strokes in adults over the age of 65 years constitute the majority of strokes. However, approximately 25% of strokes occur in patients under 65 years of age. Common causes of these strokes include hematologic disorders, connective tissue disease, substance abuse, trauma, and cardioembolic disease. About 30% of strokes in young adults have cryptogenic (undetermined) etiology [1].

Cerebrovascular disease associated with IDA has been attributed to three main mechanisms: venous sinus thrombosis from reactive thrombocytosis, arterial cerebral infarctions with possible thrombus formation, or severe hypoxia and severe anemia causing reversible neurologic deficits in the setting of atherosclerotic cardiovascular disease (ASCVD) [5, 6]. The association between IDA and stroke has been better defined in children than it has been in adults. Multiple case series have reported IDA to be associated with ischemic stroke or venous thrombosis in children (ages between 6 and 18 months) in the setting of acute viral illness as well [7]. However, very few case reports have been documented in young adults.

A 3-case report series by Akins et al. of women aged 20, 39, and 44 years described severe IDA secondary to menorrhagia associated with carotid artery thrombosis and a thrombus-induced stroke [8]. Transient ischemic attack (TIA) associated with carotid artery thromboembolism in severe iron deficiency was also described by a study conducted by Batur Caglayan et al., which hypothesized that IDA-induced microcytosis and thrombocytosis produce a hypercoagulable state [9]. It was theorized that increased blood flow to the brain causes endothelial injury, thereby leading to thrombus formation. Hypoxia from severe IDA is also hypothesized as one of the causes for stroke due to decreased oxygen-carrying capacity in the blood and, thereby, decreased delivery of oxygen to the brain [1].

Interestingly, it has been demonstrated by Huang et al. in a study that showed higher mortality within 3 years among patients with IDA at the time of admission during a first stroke from an atherosclerotic-related ischemic event [10]. This underscores the importance of identifying and correcting IDA in the setting of ischemic stroke, which can improve patient outcome.

The case of our patient who suffered an ischemic stroke at 20 years of age is intriguing because a thorough diagnostic work-up did not reveal any clear etiology apart from severe IDA. This adds to the small volume of case reports and case series that suggest IDA as a possible etiology for ischemic stroke in young adults. This is of interest as IDA is not a well-known cause of ischemic stroke in young adults. This highlights the importance of prompt diagnosis and treatment of IDA in young patients, especially after a thorough work-up has ruled out more widely recognized etiologies.
